# Adult Male with Left Arm Pain and Swelling

**DOI:** 10.5811/cpcem.2018.7.39151

**Published:** 2018-08-16

**Authors:** Jonathan Rowland, Lauren Traill, Mark Favot

**Affiliations:** Wayne State University School of Medicine, Department of Emergency Medicine, Detroit, Michigan

## CASE PRESENTATION

**Image 1:**
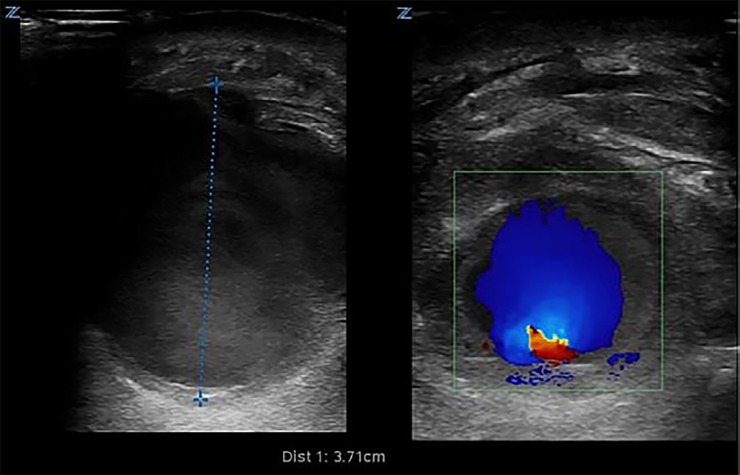
Point-of-care ultrasound using 10-5 MHz linear probe demonstrating a large 3.71 cm aneurysmal dilation of left radial artery (left). Color Doppler revealing significant pulsatile and turbulent flow (right).

**Image 2:**
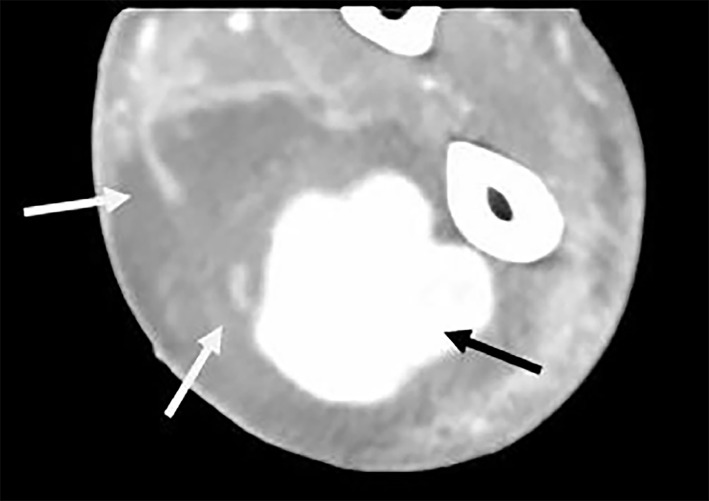
Subsequent computed tomography with intravenous contrast demonstrating a 3.2×3.4×5.2 cm radial artery aneurysm (black arrow) within the anterolateral compartment of the distal forearm, as well as diffuse cellulitis of anterior forearm soft tissue with phlegmonous changes (white arrows).

A 64-year-old male with a history of intravenous drug abuse presented to the emergency department (ED) with left arm pain and swelling for four days. Left upper extremity exam revealed diffuse swelling, erythema and tenderness in the mid-distal forearm. A point-of-care ultrasound (POCUS) was performed to characterize the suspected abscess for incision and drainage; however, imaging revealed a severely enlarged radial artery, suspected to be a pseudoaneurysm with an approximate diameter of 3.71 cm (Image 1, Video). Computed tomography of the extremity revealed an aneurysmal radial artery (Image 2). The patient was transferred to the operating room where the diagnosis was revealed.

## DIAGNOSIS

### Radial Artery Mycotic Aneurysm

Mycotic aneurysms are rare, with annual prevalence of 0.03% of injection drug users who present to the ED with complications from injection.^1^ Far more common is the development of an abscess or cellulitis at the injection site. However, due to the lethality of attempted blind incision and drainage of an aneurysm or pseudoaneurysm, it is critical to properly distinguish these from an abscess or cellulitis.

POCUS is a safe, accurate, and cost-effective modality to distinguish between these diagnoses, and can alter management by 73% when compared to clinical judgment.^2^ The sonographic appearances of an abscess and cellulitis have been well described.^3^ An abscess most commonly has an anechoic or hyperechoic spherical appearance with lobulated or irregular borders as well as possible posterior acoustic enhancement.^4^,^5^ The use of color Doppler to identify any blood flow within an anechoic structure can be a critical step to avoid a catastrophic incision into a blood-filled structure. The patient was taken to the operating room where surgeons revealed a purulent aneurysmal radial artery that was treated with ligation and debridement, parenteral antibiotics, and wound vacuum dressings.

**CPC-EM Capsule**

What do we already know about this clinical entity?

Mycotic aneurysm is a rare but serious presentation among intravenous drug abusers presenting with seemingly classic skin and soft tissue infection at injection sites.

What is the major impact of the image(s)?

Point-of-care ultrasound (POCUS) of suspected abscesses prior to incision and drainage is an effective method of securing this diagnosis and ensuring you are not incising something else.

How might this improve emergency medicine practice?

This case further supports the use of POCUS in helping emergency providers to avoid rare but potentially catastrophic complications during routine abscess evaluation and management.

Documented patient informed consent and/or Institutional Review Board approval has been obtained and filed for publication of this case report.

## Supplementary Information

Video Radial artery mycotic aneurysm.

## References

[1] Tsao JW, Marder SR, Goldstone J (2002). Presentation, diagnosis, and management of arterial mycotic pseudoaneurysms in injection drug users. Ann Vasc Surg.

[2] Adhikari S, Blaivas M (2012). Sonography first for subcutaneous abscess and cellulitis evaluation. J Ultrasound Med.

[3] Subramaniam S, Bober J, Chao J (2016). Point-of-care ultrasound for diagnosis of abscess in skin and soft tissue infections. Acad Emerg Med.

[4] Gaspari RJ, Blehar D, Polan D (2014). The Massachusetts Abscess Rule: a clinical decision rule using ultrasound to identify methicillin-resistant *Staphylococcus aureus* in skin abscesses. Acad Emerg Med.

[5] Nelson CE, Chen AE, Bellah RD (2018). Ultrasound features of purulent skin and soft tissue infection without abscess. Emerg Radiol.

